# PolyBall: A new adsorbent for the efficient removal of endotoxin from biopharmaceuticals

**DOI:** 10.1038/s41598-019-45402-w

**Published:** 2019-06-20

**Authors:** Sidharth Razdan, Jee-Ching Wang, Sutapa Barua

**Affiliations:** Department of Chemical and Biochemical Engineering Missouri University of Science and Technology, Rolla, MO 65409 USA

**Keywords:** Chemical engineering, Nanoparticles

## Abstract

The presence of endotoxin, also known as lipopolysaccharides (LPS), as a side product appears to be a major drawback for the production of certain biomolecules that are essential for research, pharmaceutical, and industrial applications. In the biotechnology industry, gram-negative bacteria (*e*.*g*., *Escherichia coli*) are widely used to produce recombinant products such as proteins, plasmid DNAs and vaccines. These products are contaminated with LPS, which may cause side effects when administered to animals or humans. Purification of LPS often suffers from product loss. For this reason, special attention must be paid when purifying proteins aiming a product as free as possible of LPS with high product recovery. Although there are a number of methods for removing LPS, the question about how LPS removal can be carried out in an efficient and economical way is still one of the most intriguing issues and has no satisfactory solution yet. In this work, polymeric poly-ε-caprolactone (PCL) nanoparticles (NPs) (*d*_*P*_ = 780 ± 285 *nm*) were synthesized at a relatively low cost and demonstrated to possess sufficient binding sites for LPS adsorption and removal with ~100% protein recovery. The PCL NPs removed greater than 90% LPS from protein solutions suspended in water using only one milligram (mg) of NPs, which was equivalent to ~1.5 × 10^6^ endotoxin units (EU) per mg of particle. The LPS removal efficacy increased to a higher level (~100%) when phosphate buffered saline (PBS containing 137 mM NaCl) was used as a protein suspending medium in place of water, reflecting positive effects of increasing ionic strength on LPS binding interactions and adsorption. The results further showed that the PCL NPs not only achieved 100% LPS removal but also ~100% protein recovery for a wide concentration range from 20–1000 μg/ml of protein solutions. The NPs were highly effective in different buffers and pHs. To scale up the process further, PCL NPs were incorporated into a supporting cellulose membrane which promoted LPS adsorption further up to ~100% just by running the LPS-containing water through the membrane under gravity. Its adsorption capacity was 2.8 × 10^6^ mg of PCL NPs, approximately 2 -fold higher than that of NPs alone. This is the first demonstration of endotoxin separation with high protein recovery using polymer NPs and the NP-based portable filters, which provide strong adsorptive interactions for LPS removal from protein solutions. Additional features of these NPs and membranes are biocompatible (environment friendly) recyclable after repeated elution and adsorption with no significant changes in LPS removal efficiencies. The results indicate that PCL NPs are an effective LPS adsorbent in powder and membrane forms, which have great potential to be employed in large-scale applications.

## Introduction

In biotechnology industries, gram-negative bacteria are widely used for the production of therapeutic biomolecules including proteins, peptides, and nucleic acids^1–6^. These biomolecules are recovered by cellular rupturing that leads to the release of a large number of bacterial cell-wall components containing endotoxins, also known as lipopolysaccharides (LPS)^7–9^. When the LPS contaminated products are administered to animals or humans even in small quantities (0.05–0.1 ng/ml), a systemic inflammatory reaction can occur, leading to multiple pathophysiological effects, such as septic shock, tissue injury, and lethality^10,11^. Removing undesirable LPS from solutions is thus an important aim in the pharmaceutical industry and in clinical practice. Conventional treatments such as coagulation and membrane filtration are adequate for removing bacteria cells and debris but not effective for removing dissolved endotoxins to a significant extent. Therefore, it is highly desirable and also the focus of this project to develop a biodegradable and inexpensive means that can tackle both aspects of LPS removal.

A number of approaches have been developed and typically utilized to reduce LPS concentration in pharmaceutical solutions and therapeutic products or in purified water^8,12–33^. These approaches employ activated carbon^34,35^, gel filtration chromatography^12–15^, ion exchange or size exclusion chromatography^16–20^, sucrose gradient centrifugation^36–38^, Triton X-114 phase separation^39–41^, ultrafiltration^21,22^, microfiltration^21,22^ and affinity adsorbents^23–28^, functionalized with L-histidine^42^, poly(ethylene imine) (pEI)^23^, poly-ε-lysine, poly(γ-methyl L-glutamate), or polymyxin B^8,29–33^. and chemical means such as ozonation and chlorination^35,43^. More recently, nanoparticle (NP)-based methods have also been attempted and shown great promise^44–46^. Polymyxin B capped silver (Ag) NPs have been used to remove LPS from aqueous solutions, up to 97% efficiency, based on the ionic interaction between the cationic peptide on Polymyxin B and the anionic phosphate on Lipid A of LPS^44^. Surface modified iron oxide (Fe_3_O_4_) gold (Au) core-shell nanoflowers (NFs) have been explored for simultaneous reduction and detection of LPS as alternatives to classical methods of endotoxin sensing^47^. Also, NPs with a polystyrene core and a polyglycidyl methacrylate shell have been synthesized and further modified with amine-based, amino acid based, pEI, tetracaine, or Polymyxin B ligands for LPS removal from water and salt solution^46^. The parent particles modified with amine-based (ethylene diamine, hexamethylene amine, and dodecyl diamine) and pEI ligands showed significant LPS removal efficiency around 90% from both water and salt solution, whereas those modified with tetracaine, amino acid lysine, and amines (histamine and tryptamine) showed a higher LPS removal efficiency from water, also around 90%, than from salt solution^46^. While showing great promise, these approaches at present still have their shares of limitations and disadvantages in terms of cost, efficiency, degradability, side effects, and/or accompanying toxicity brought by the reagents. For examples, the methods utilizing porous functionalized NPs are reasonably effective in reducing the LPS concentration; however, their operations are relatively expensive due to the use of high-pressure equipment that adds significant cost to downstream purification and are contingent on the slow processes of intraparticle diffusion and solute retention on the binding sites^48–50^. Polymyxin B, a polypeptide antibiotic, can also cause neurotoxicity and nephrotoxicity.

A key step forward with the NP-based approach is to establish a high throughput, low-cost method that is not subject to high pressure-drop limitation, slow solute transport, or accompanying toxicity. To this end, poly-ε-caprolactone (PCL) NPs without any modification have been manufactured in the PI’s laboratory, which are non-porous solid adsorbent nanoparticles with solute binding sites situated on the particle surface. The NPs were found to be capable of adsorbing and removing LPS from protein solutions at efficiency up to 100%. Their prospects for technological application were further substantiated by the processing feasibility of incorporating PCL NPs into membrane filters and high LPS reduction and removal from biological solutions using cellulose membranes embedded with PCL NPs. In either powder form or in a spread bed of a flat sheet membrane, PCL NPs offer high adsorption capacity per unit mass of the adsorbents. Since PCL and cellulose are both low-cost biocompatible polymers^51–53^, the use of such PCL NP-embedded membranes represents a novel LPS separation system that requires low capital costs but provides desirable ease of manufacturing, excellent performance, disposability, and biodegradability.

## Materials and Methods

### Synthesis of PCL nanoparticles

PCL NPs were synthesized by the solvent evaporation method which utilized high–speed homogenization and sonication, followed by solvent evaporation, centrifugation to remove surfactants, and then lyophilization^54–58^. A PCL solution at a concentration of 10 mg/ml in ethyl acetate was injected using a syringe pump to a 1% w/v polyvinyl alcohol (PVA) solution prepared with reverse osmosis (RO) water. The mixture was homogenized by using a homogenizer rotating at 3000 rpm while being placed in a sonication bath. Ethyl acetate was removed by stirring the mixture at 300 rpm for two days. The obtained particles were washed five times using RO water and centrifuged for 30 minutes at 10,000 rcf. The resulting products were freeze-dried, weighed, and stored at 4 °C until further use. To test the effects of cationic charges on bare PCL NP, 10 mg of freeze-dried PCL NPs were coated with cationic PLL solution by incubating with 1 ml of 0.1% (w/v) PLL (Sigma) for 1 h. Post incubation the particle suspension was centrifuged for 30 min at 16,000 rcf and the supernatant was separated to obtain positively charged PLL coated PCL NPs.

### Characterization of PCL nanoparticles

The morphology of PCL NPs was observed using Hitachi S-4700 scanning electron microscope (SEM) at an accelerating voltage of 15 kV. Samples were sputter coated with Denton Au/Pd coater before inserting it into the microscope. The average PCL particle size was measured by analyzing the SEM images using the ImageJ software (version 1.51w). The average particle size was reported as mean ± standard deviation (SD) based on the diameters of 200 randomly selected particles. The hydrodynamic size and surface charge of NPs were characterized by dynamic light scattering (DLS) and zeta $(\zeta )$ potential measurements, respectively using Malvern NanoZS90 Zetasizer. The hydrodynamic diameter of PCL NPs was measured at 25 °C using He-Ne Lasers at 90° scattering angle. The size distribution was obtained based on three independent experiments utilizing 100 successive runs. Zeta potential values were reported based on three independent experiments with each experiment utilizing 15 successive runs and the results were reported as millivolts (mV) ± SD.

### Adsorption studies

*Escherichia coli* O111: B4 LPS (Sigma Aldrich) was used to study the adsorption capacities of PCL NPs in aqueous solutions in batch experiments. Initial experiments were carried out using a constant LPS concentration (150 µg/ml) treated with different PCL concentrations (0.1, 25, 50, 100, 200, 300, 400, 500, 750 and 1000 μg/ml) in: (i) RO water (pH ~6); (ii) phosphate buffered saline (PBS; 150 mM, pH ~7.4); (iii) bovine serum albumin (BSA) solutions in water and PBS; (iv) Trastuzumab (TTZ; Genentech) solutions in water and PBS; (v) Fibrinogen (Alfa Aesar) in PBS and (vi) Human Hemoglobin (MP Biomedicals) in PBS. The composition of PBS is as follows: 137 mM NaCl, 10 mM phosphate, and 2.7 mM KCl at 25 °C. The LPS binding capacity to PCL NPs was analyzed using Bodipy (BOD) fluorescence displacement assay technique^58,59^. BOD is a fluorescent molecule that quenches its fluorescence intensity (F.I.) when it binds to LPS. The F.I. of BOD was used to determine the LPS concentration in solution using a known standard calibration curve (Figs S1, S2). The F.I. measurements were carried out using a microplate reader (BioTek). Excitation and emission wavelengths for BOD were 485/20 and 528/20 nm, respectively. RO water was used as a negative control. The background fluorescence intensities were subtracted to avoid any interferences. The percentage (%) LPS removal by PCL NPs from water and PBS was calculated using Eq. (1):

1 $${\rm{ \% }}\,LPS\,removal=\frac{F{I}_{BOD.LPS.PCL}-F{I}_{BOD.LPS}}{F{I}_{BOD}-F{I}_{BOD.LPS}}\times 100$$

where $F{I}_{LPS},\,F{I}_{BOD.LPS}$, and $F{I}_{BOD.LPS.PCL}$represent the F.I. of BOD alone, LPS mixed with BOD, and LPS mixed with BOD and PCL NPs, respectively.

The % LPS removal by PCL NPs from protein solutions was calculated using Eq. (2):

2 $${\rm{ \% }}\,LPS\,removal=\frac{F{I}_{BOD.Protein.LPS.PCL}-F{I}_{BOD.Protein.LPS}}{F{I}_{BOD}-F{I}_{BOD.Protein.LPS}}\times 100$$

where $F{I}_{BOD},\,\,F{I}_{BOD.Protein.LPS}$, and $F{I}_{BOD.Protein.LPS.PCL}\,\,$represent the F.I. of BOD alone, LPS mixed with BOD and protein, and LPS mixed with BOD, protein and PCL NPs, respectively.

The adsorption capacity at equilibrium (*qe*) was evaluated using the following equation:

3 $${q}_{e}=\frac{({C}_{0}-{C}_{e})}{W}\times V$$

where ${C}_{0},\,\,{C}_{e},\,W$, and $V$ represent the initial LPS concentration (μg/ml), the corresponding LPS concentration at equilibrium (μg/ml), the PCL NP’s mass amount (mg), and the solution volume (ml), respectively. The isotherm data were fitted into the linear Freundlich model equation (4) to describe the adsorption equilibria:

4 $$ln{q}_{e}=lnK+\frac{1}{n}ln{C}_{e}$$

Where, ${q}_{e}$, $K$,$\,n$, and ${C}_{e}$ represent the adsorption based binding capacity (μg LPS per mg PCL NPs), Freundlich (binding affinity) constant (μg LPS per mg PCL NPs), Freundlich exponent and equilibrium LPS concentration (μg LPS/ml solution), respectively.

### Protein recovery

Protein recovery in LPS spiked sample solutions was quantified using BCA assay kit (Pierce). The absorbance at 562 nm was measured in a microplate reader (BioTek). Different concentration of BSA, TTZ, fibrinogen and human hemoglobin were used for plotting the individual protein’s standard curves (Fig. S3). All assays were performed by the manufacturer’s instructions.

### Effects of buffer and pHs on LPS removal

The effect of different buffers on LPS binding efficiency was analyzed by interacting a fixed PCL NP concentration (1000 µg/ml) with a constant LPS concentration (150 µg/ml) prepared using different buffer solutions recipes (Table S1) each having fixed ionic strength of 100 mM (0.1 M). Six different buffer pH values from 2.8–9.6 were tested. Glacial acetic acid was used to obtain a pH value of 2.8. Phosphate buffers were prepared from monobasic and dibasic salts of 0.2 M sodium phosphate to obtain pH values of 5.8, 6.8 and 8^60–62^. PBS of sodium bicarbonate were used to prepare pH 7.4 and 9.6 buffers, respectively^60–62^.

### Effect of salt concentration on protein recovery

To investigate the effect of salt concentration on % protein recovery, 1000 μg/ml of each BSA and TTZ were spiked with 150 μg/ml of LPS in the different range of PBS concentrations: 0, 0.1, 1, 10, 100 and 150 mM. Protein concentrations were measured before and after LPS spiking and used to further calculate the % protein recovery.

### PCL NP regeneration studies

PCL NP suspension was interacted with fixed LPS concentration (270 µg/ml) in RO water and then centrifuged to obtain the supernatant which was reacted with BOD to calculate the percent LPS removal efficiency using equation (1). The PCL NP pellet was resuspended in 0.2 N sodium hydroxide (NaOH) solution for 2 h and then centrifuged to remove the NaOH supernatant. The PCL NP pellet was washed five times using RO water before reusing it again for LPS binding. This regeneration cycle was repeated three times to measure any loss in LPS binding efficiency for PCL. The LPS removal efficiency of PCL NPs after each washing cycle was measured using the BOD fluorescence assay.

### Synthesis of cellulose acetate (CA) membrane

The CA membranes with or without PCL NPs were prepared by a non-solvent induced phase separation process^63^. A casting solution was prepared by dissolving 10 wt.% each of CA and 5 wt.% Pluronic F127 in dimethyl sulfoxide (DMSO) (control). For membranes with NPs, 1 wt.% of PCL NPs was dispersed in the casting solution under vigorous stirring (1100 rpm) at 50 °C for 1 h to allow homogenous mixing and the solution was then left for 2 h to allow the complete release of bubbles. The final solution was cast on a casting plate and then immersed in RO water coagulation bath for 30 min. Finally, the water wet membrane was immersed in 30% glycerol (plasticizer) for 15 min, which in addition to improving the mechanical properties also help in dry storage of the membrane for at least 300 days with no major loss in membrane flux and removal properties^64^. The mass loading of PCL NPs in CA membranes was quantified by comparing the weights of 10 randomly freeze-dried membrane pieces of the same area (1.8 cm^2^) before and after adding the NPs. The measured weight difference of the membranes with and without NPs is the mass of PCL NPs added to the membrane and was used to calculate LPS removal per unit mass of PCL NPs.

### Microscopy and microanalysis

The CA membranes with or without PCL particles were dried using the freeze-fracture method^65^. Samples were attached to an SEM stub and sputter coated with Denton Au/Pd coater. The membrane surface and cross sections were imaged using the Hitachi S-4700 SEM operated at 3 kV. The membrane surface and cross-sectional morphology, pore size, and thickness were analyzed using ImageJ software (version 1.51w). The average membrane pore size and thickness were based on 100 randomly selected pores and points from different images. The results were reported as average ± standard deviation (SD). The presence of PCL NPs in the membrane was further verified using fluorescein isothiocyanate (FITC; 1 wt%) incorporated PCL NPs and fluorescence microscopy (Zeiss) equipped with 470 ± 40/525 ± 50 nm excitation/emission filters.

### Permeation studies

The measurement of permeation flux was conducted using a custom-made membrane test apparatus (Fig. S4). The apparatus was made of two polyvinyl chloride flow pipes that hold the membrane in between like a sandwich. Each flow pipe is 1.5 cm wide. The top and bottom pipes are 20 cm and 10 cm long, respectively. The membrane area was 1.8 cm^2^. In each experiment, a volume of 20 ml water or solution was fed to the top pipe in a batch setup and flowed through the membrane by gravity. For LPS mixed water, a concentration of 270 μg/ml LPS in 20 ml water was used. Water was collected from the end of the bottom pipe. The water volume was measured at 1 h interval for 8 h to calculate the change in water flux.

### Quantification of LPS removal using PCL NPs in CA membranes

The determination of LPS removal by CA membranes with or without PCL particles was also carried out by BOD fluorescence displacement assay technique^58,59^ and the apparatus introduced above. A volume of 20 ml RO water containing 270 μg/ml of LPS was fed to the top flow pipe to flow through a sandwiched membrane by gravity. A fixed volume (277 µL) of the LPS feed and the permeate was collected every hour until 8 h. The samples were mixed with BOD (262.11 µg/ml) and the F.I. of BOD was measured using a plate reader (BioTek). The percent (%) LPS removal was calculated using equation (5),

5 $$Cumulative\,{\rm{ \% }}\,LPS\,removal=(1-\frac{F{I}_{BOD}-F{I}_{BOD.LPSinpermeate}}{F{I}_{BOD}-F{I}_{BOD.LPSinfeed}})\times 100$$

where $F{I}_{BOD}$, $F{I}_{BOD.LPSinpermeate}$, and $F{I}_{BOD.LPSinfeed}$ are the F.I.s of BOD alone, BOD mixed with LPS in permeate, and BOD mixed with LPS in the feed solution, respectively. Each value used here was based on triplicate measurements from three independent experiments. The mean differences and standard deviations were also evaluated.

### Calculation of LPS removal efficiency per unit mass and surface area of PCL NPs

The LPS removal efficiency per unit mass and surface area were calculated for PCL NPs used in powder form or in the CA membrane. This required the calculation of the number of PCL NPs per unit solution volume using equation (6).

6 $$Number\,of\,PCL\frac{NPs}{ml}=\frac{6c\times {10}^{12}}{\rho \pi {d}_{p}^{3}}$$

where $c$ is the concentration of particles in solution in g/ml, $\rho$ is the density of PCL NPs in g/ml, and ${d}_{p}$ is the particle diameter in µm. The mass loading of PCL NPs entrapped in a CA membrane was measured from the mass difference of the freeze-dried CA membranes with and without NPs. The LPS removal efficiency per unit cm^2^ and per unit milligram of NPs was calculated based on the mass of LPS in the feed solution and the maximum % LPS removal.

## Results

### Removal of LPS from water and PBS using PCL NPs in powder form

**Figure 1:**
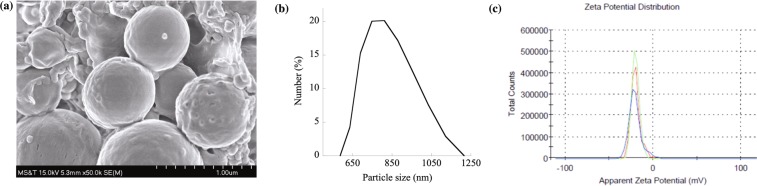
Characterization of PCL NPs. (**a**) An SEM image of PCL NPs at 50,000 X magnification. (**b**) Plot showing size distribution of PCL NPs. (**c**) Zeta potential of PCL NPs in water. Three colors indicate three independent runs.

**Figure 2:**
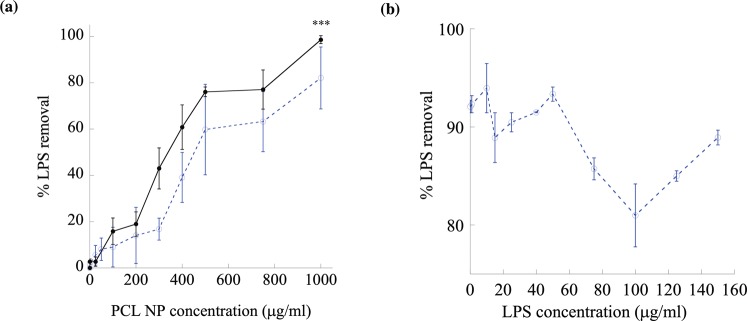
The LPS removal efficiency of PCL NPs from water and PBS. (**a**) The percent (%) LPS removal from water (open circles, •; dotted line) and PBS (filled, solid circles, •; solid line) following adsorption on PCL NPs. *** indicates the p-value < 0.005 showing a statistically significant difference between % LPS removal in water and PBS. A fixed LPS concentration of 150 μg/ml was used in this study. (**b**) Water containing low (0.1 μg/ml) to high (150 μg/ml) LPS concentrations were treated with 1000 μg/ml of PCL NPs that gives ~95% LPS removal.

The size of PCL NPs was observed to be $780\pm 285\,\,nm$ in diameter by analyzing SEM images (Fig. 1a) and DLS technique (Fig. 1b), which, relatively speaking, is fairly uniform with a low level of dispersity in size. The surface morphology shows that the NPs were of highly spherical shape and their surfaces appeared to be closely packed without apparent pores leading into the interior of the particles. The $\zeta$ potential of PCL NPs was found to be $-20\pm 5\,mV$ in water (Fig. 1c) indicating a stable dispersion that resists aggregation. LPS adsorption tests were carried out with PCL NPs in both water (open circles; dotted line; Fig. 2a) and PBS (filled, solid circles; solid line; Fig. 2a) where initially the concentration of PCL NPs was systematically varied from 0 to 1000 μg/ml in both cases at a fixed LPS spiked concentration of 150 μg/ml and then the concentration of PCL NPs was fixed and the concentration of LPS was varied from 0.1 to 150 μg/ml in RO water. It was clear and important to note first that PCL NPs were effective in adsorbing and removing LPS from solutions regardless of the presence or absence of salts (PBS). In general, the removal efficiency of LPS by PCL NPs increased with increasing PCL NP concentration, which was to be expected due to increasing numbers of active sites available in the system for binding to LPS. The maximum level of LPS removal achieved was 98% when the PCL NP concentration of *c* = 1000 μg/ml was used under the positive influence of salts. Without salts, the LPS sequestration from water was only ~1.8% at a low NP concentration of 0.1  μg/ml and increased to 9% and 82% when the NP concentration became 100 and 1000 μg/ml, respectively. The result at *c* = 1000 μg/ml was used to evaluate the LPS removal efficiency with varying LPS concentrations of 0–150 μg/ml in water (Fig. 2b). The maximum LPS removal efficiency was ~95%, which was approximately $\sim 2040\,endotoxin\,\,units\,(EU)/c{m}^{2}$ or ~1.3 × 10^6^ EU/mg of PCL NPs (Table S2).

Across the whole concentration range, the LPS adsorption increased with the addition of salt (PBS; pH 7.4) to water (solid circles; Fig. 2a). This positive effect was clearly exhibited by the data beyond any uncertainty of measurement and indicated that increased ionic strength by the addition of salts resulted in higher LPS adsorption on the PCL NP surface. It is possible that at this high salt concentration (150 mM PBS) a strong interaction between water molecules and salts creates a shielding off effect leaving less water available for the induction of interactions between LPS and PCL. This behavior is consistent with the previously published literature^66–71^. Another possible explanation could be an electrostatic screening effect that reduces the repulsive interaction between two moieties carrying the same type of charges. Although both LPS and PCL can generally be considered hydrophobic molecules, the former exhibits a net negative charge due to its phosphate groups^33^ and the latter also possesses partial negative charges in its carbonyl oxygen atoms. The repulsion between these negative charges can be understood to be weak relative to the van der Waals and hydrophobic binding^46^ between the two massive molecules and hence unable to impede the overall binding interaction and adsorption between LPS and PCL. However, this repulsion can be further weakened, thereby giving rise to stronger binding interaction and heightened adsorption, by the presence of salt ions in proximity to the negative charges that shield their like-charge interactions.

### Removal of LPS from protein solutions using PCL NPs

**Figure 3:**
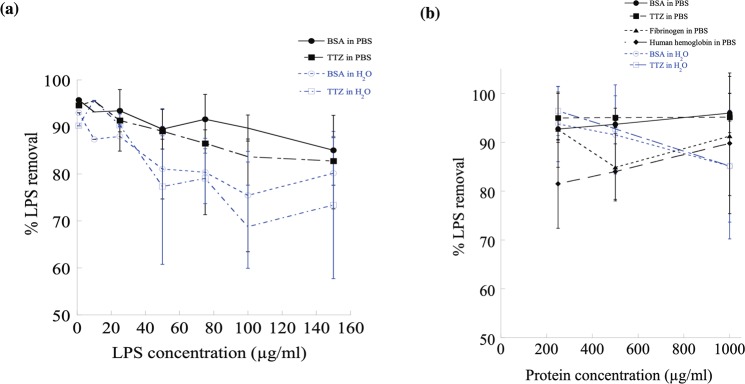
The LPS removal efficiency of PCL NPs from protein solutions. (**a**) Increasing LPS or (**b**) protein concentrations have no significant effect on the % of LPS removal from protein solutions prepared in water and PBS. Symbols •, ο, ▪, ▲, ♦ indicate LPS containing BSA solutions in PBS, BSA in water, trastuzumab (TTZ) solutions in PBS, TTZ in water, Fibrinogen in PBS and Human hemoglobin in PBS respectively.

To study the effectiveness of PCL NPs on removing LPS at the common contamination level from 0–150 μg/ml in biopharmaceutical solutions, two protein solutions were investigated. For this purpose, BSA and TTZ protein solutions (~1 mg/ml) in PBS of pH 7.4 and RO water containing either low or high levels of LPS were exposed to 1000 μg/ml PCL NPs (Fig. 3a). It is worth noting that the % LPS removal was higher (90–100%) in PBS (solid lines, Fig. 3a) than in water (dotted lines, Fig. 3a) indicating that PCL NPs were effective in removing LPS from pharmaceutical protein formulations^72^. We further tested the effects of protein concentration on LPS removal by analyzing four different protein solutions spiked with a fixed concentration (150 μg/ml) of LPS (Fig. 3b). Increasing protein concentrations from 250 to 1000 μg/ml did not alter the ~90% LPS removal efficacy in PBS (solid lines, Fig. 3b) by PCL NPs (1000 μg/ml). In the case of BSA and TTZ in water, the % LPS removal dropped from 95% to ~ 80% with the increment in protein concentrations. This reduction of LPS binding on PCL NPs at high protein concentrations in water could be either due to: (i) exchange of low affinity of the highly abundant protein binding with the NP surface by the lower abundance of LPS with a higher affinity for the NP surface; and/or (ii) formation of large aggregates between LPS-protein molecules desorbing LPS from the NP surface. In PBS, the % LPS removal from protein solutions was higher than that in water presumably due to more stable LPS-PCL NP complex formation surrounded by ions in bulk solution. On a preparative scale, an important indicator of desirable properties from such NP adsorbents is the adsorption capacity per unit mass. For this purpose, the equilibrium LPS adsorption capacity of PCL NPs was calculated up to $1.4\times {10}^{6}\,EU/mg$ with ~100% LPS removal capacity from BSA, TTZ, fibrinogen and human hemoglobin solutions in PBS of pH 7.4 (Tables S3–S6).

### LPS adsorption behavior on PCL NPs

**Figure 4:**
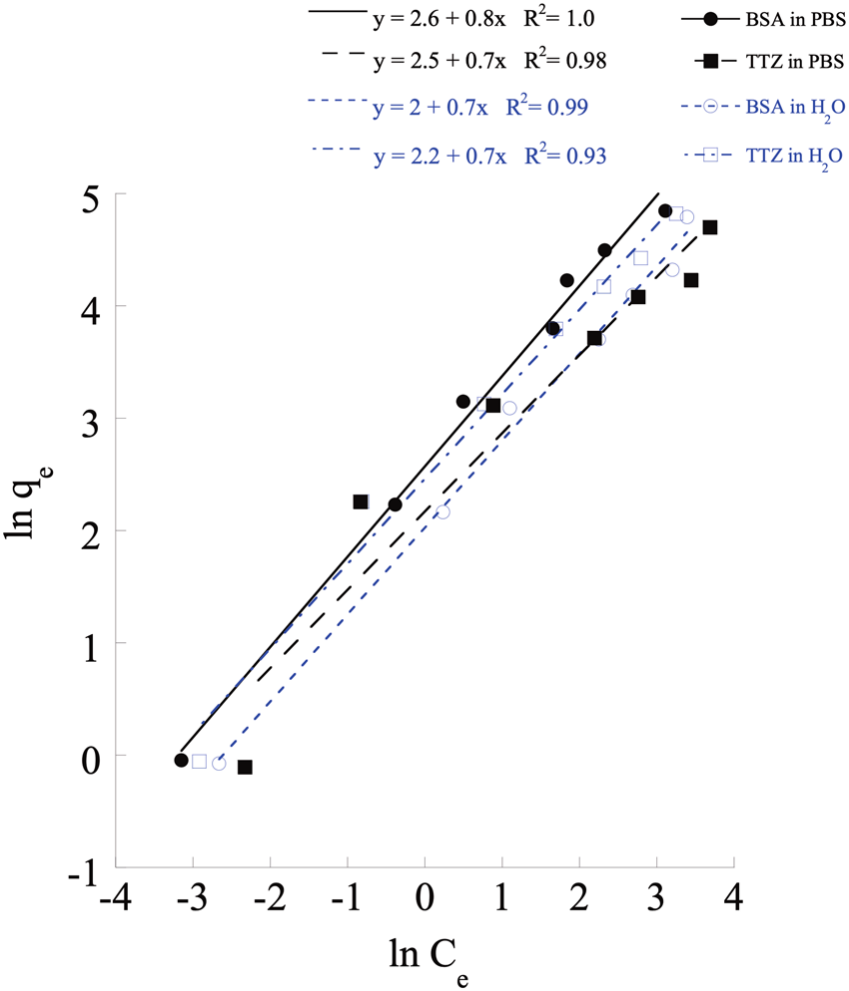
Freundlich adsorption isotherm fitting of LPS removal by PCL NPs from BSA and TTZ solutions in water and PBS.

Based on the experimental data of LPS binding on PCL NPs, binding-dependent parameters were calculated using the Freundlich isotherm model that rationalizes the contribution of favorable adsorption on the NP surface. The experimental data fit the Freundlich model (R^2^ > 0.98) where the slope $\frac{1}{n}$ accounts for the intensity of adsorption and intercept, $K$ measures the binding affinity (μg LPS/mg PCL NPs) (Fig. 4). $n > 1$ represents favorable adsorption associated with multilayer LPS formation on the PCL surface^73,74^. From Table S7, it can be seen that the binding intensity (n) values vary from 1.1–1.4 thus indicating that the NPs have favorable LPS binding adsorption performance for all tested conditions^75^. The binding affinity constant, *K* was found to vary between 9.5–11.7 μg LPS/mg PCL NPs ($\sim {10}^{5}{\textstyle \text{-}}{10}^{6}\,{\rm{E}}{\rm{U}}/{\rm{m}}{\rm{g}}\,$) depending on the solution (water and PBS) and protein types (BSA and TTZ). The *K* values were compared with previously reported sorbents^76–79^ which indicated that PCL NPs were 10 to 40 log orders of magnitude better in LPS binding capacity than most of the commonly used adsorbents such as Polymyxin B conjugated cellulose microspheres and Histidine immobilized silica gels, among others^76–79^. To tease out the interactions between LPS and PCL NPs, the NPs were coated with a cationic polymer, PLL (Fig. S5). The PLL coated PCL NPs showed a significant decrease in % LPS removal from 80% to 60% in water and from 100% to 20% in PBS. These findings reassert the selective hydrophobic interactions between LPS and PCL NPs.

## Protein Recovery

**Figure 5:**
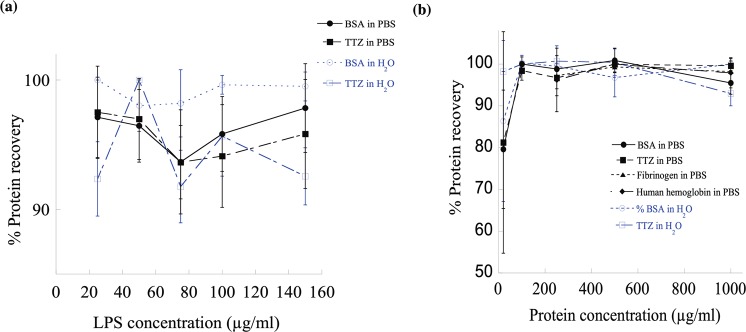
Percentage of protein recovery as a function of (**a**) protein concentrations and (**b**) LPS concentrations. The amount of PCL NPs used was 1000 μg/ml.

Most biopharmaceutical purification processes suffer from product loss. Protein recovery is as important as LPS removal to reflect an interaction of the protein with LPS binding sites. Figure 5 shows the results of protein recovery at varying (**a**) LPS and (**b**) protein concentrations. As it is seen that protein recoveries were close to 100% for a wide range of LPS (0–160 μg/ml) and protein (0–1000 μg/ml) concentrations. These results further confirm the selectivity of PCL NPs for LPS while showing 100% protein recovery.

### Effect of pH on LPS removal in different buffer conditions

**Figure 6:**
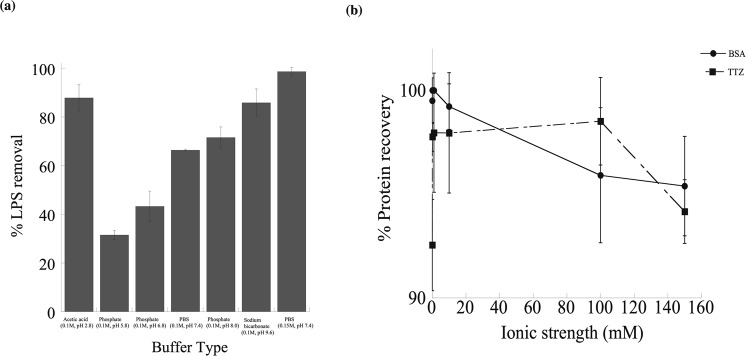
The effects of pH and salt concentrations on LPS removal by PCL NPs. (**a**) The effect of pH and buffers on the % LPS removal. Four different types of buffers (acetic acid, phosphate, PBS and sodium bicarbonate) covering pH range from 2.8–9.6 were used. (**b**) Dependence of protein recovery on salt concentrations in LPS and PCL NP systems. Solid line with filled, solid circles (•) represents BSA and the dotted line with filled, solid squares (▪) indicates TTZ.

The percentage of LPS removal was predicted to be dependent on the changes in buffer pH (Fig. 6a). LPS binding on PCL NPs show reasonably strong dependence on pH for different buffers of variable pHs. The ionic strength for all buffers was maintained constant at 100 mM (0.1 M). At the pH of 2.8, *i*.*e*., near and below the isoelectric point (pI 2) of LPS^80^, the binding of LPS with PCL NPs increased close to ~90% possibly due to low LPS solubility near the pI and high hydrophobic interactions between non-polar LPS and PCL resulting in increased LPS removal from the solution. On the other hand above the pI of LPS, at pHs between 5.8 and 8, average LPS removal efficiencies were found to be increased from 30% up to 90% in an alkaline buffer pH of 9.6. The enhancement in LPS removal at high pH is most likely due to hydrophobic interactions between non-polar LPS and PCL NPs that segregate the polar ions and water molecules and minimizes the area of contact between polar and non-polar molecules in the solution^81^. The phase separation of LPS was further enhanced up to ~99% by PBS of higher ionic strength (0.15 M, pH 7.4) driving the self-assembly of LPS-PCL NP hydrophobic effects. In summary, PCL NPs can operate in acidic to neutral conditions (pH 2.8 to pH 9.6). The highest LPS removal (~100%) was found in PBS of pH 7.4 followed by > 85% recovery in acetic acid and sodium bicarbonate buffer of pH 2.8 and 9.6, respectively.

### Effect of salt concentration on protein recovery

Figure 6b shows that the % protein recovery is almost linear that varies between 90 to 100% with the change in salt concentrations indicating that the ionic strength has a little effect on protein recovery in our LPS-PCL NP system. At low salt concentrations extrapolated from zero salt concentration (water), the recovery was >90% for both BSA and TTZ which were increased further up to ~100% at higher salt concentrations (150 mM). These results indicate that the low affinity of proteins towards PCL NPs both in the absence and presence of solution ions. The mutual interactions between LPS and PCL NPs keep protein away in the bulk phase. At higher ionic strength, it is possible that free ions rearrange themselves into certain configurations around LPS-PCL NP complexes and proteins that promote increased retention of proteins in the mixture and thus slightly decrease the protein recovery to ~95%.

### PCL NPs were regenerated to remove LPS

**Figure 7:**
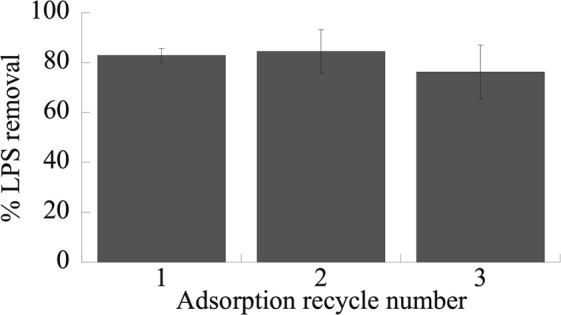
PCL NP regeneration. LPS removal efficiency after PCL NPs is regenerated three times by desorbing LPS from the NPs using 0.2 N NaOH and testing for LPS adsorption/removal.

PCL NPs were regenerated by breaking LPS-PCL complexes in RO water which makes the LPS removal process more efficient and scalable (Fig. 7). NaOH was used to regenerate the PCL NPs that exchanged off LPS for the hydroxide ($O{H}^{-1}$) ion in the caustic solution which is well-known to desorb LPS from chromatography resins and particles quite effectively^82–84^. The collected PCL NPs were re-dissolved off the $O{H}^{-1}$, and this is facilitated by the 2 h contact time. A high LPS (EU/ml) recovery (~80%) was observed over the course of three regeneration cycles. An average LPS recovery of >$2\times {10}^{6}$ EU/ml was obtained per regeneration cycle when LPS bound PCL particles were reacted with 0.2 N NaOH for 2 h and then washed using RO water before being reused for LPS binding again. Overall, the LPS removal efficiency of PCL NPs nearly had any change after three rounds if adsorption, elution, and reuse.

### PCL NPs were embedded in CA membranes

**Figure 8:**
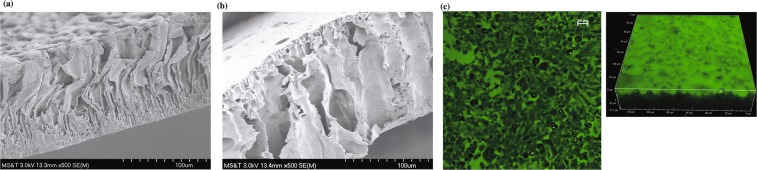
Characterization of PCL NP embedded filter. SEM images of the cross-sections of membranes prepared from (**a**) CA membrane, scale bar = 100 μm, (**b**) CA membrane with PCL NPs in low magnification, scale bar = 100 μm and (**c**) Fluorescence microscopic images of fluorescein dye encapsulated PCL NPs in membranes in high magnification.

The cross-sections of CA membranes were obtained by SEM (Fig. 8a) and compared with and without NPs. The original CA membrane exhibited a thickness of 116 ± 2 μm and a relatively homogeneous macrostructure with a distinctive dense layer near the surface (Fig. 8a). Simply from the point of view of the ratio (~100) between the membrane thickness and the particle diameter, the presence of PCL NPs could be expected to have a great impact on the structural and transport properties of the membrane. Indeed, the CA membrane with PCL NPs showed a seemingly more uniform cross-sectional structure with no unique layer (Fig. 8b), which was revealed fluorescence microscopy to contain green dye-labeled spherical PCL particles on the flat surface of the membrane (Fig. 8c). The cavities in the PCL embedded membrane were found to be noticeably larger than those in the original CA membrane as visualized from the SEM images of their cross-sections (Fig. 8a,b). While the incorporation of PCL NPs in the membrane appeared not to affect the pore opening size as there was only a slight change from 0.16 ± 0.05 μm to 0.17 ± 0.05 μm (Fig. S6), it has much greater impact on the membrane’s macro-void cross-sectional morphology as it changed from a narrow, tortuous, and flaky pore structure (Fig. 8a) to a broad, straight, and finger-like pore structure (Fig. 8b)^63,85–90^. PCL NPs also increased the membrane thickness by more than 13%, from 116 ± 2 μm to 132 ± 12 μm (Table S8).

### Permeation of water using CA membranes without and with PCL NPs

**Figure 9:**
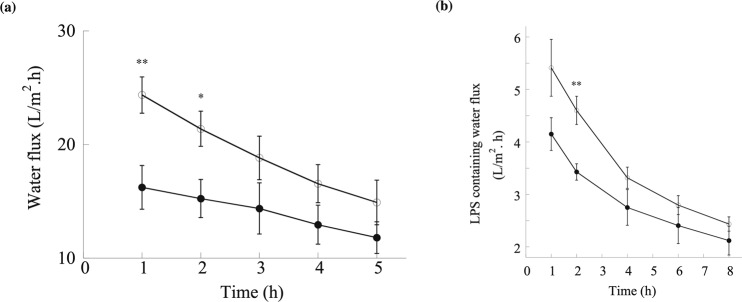
The water flux performance of CA membrane (open circles; ○) and CA membrane impregnated with PCL NPs (filled, solid circles; •) (**a**) in the absence of LPS and (**b**) in the presence of LPS. The flow rates were measured under gravity. Error bars represent standard deviations from three independent experiments. * and ** indicates p values of 0.03 and 0.01, respectively, representing statistically significant differences between the CA membrane and PCL NPs in CA membrane.

The measurement of water flux driven by gravity-flow through CA membrane was illustrated in Fig. S4, which did not require any pumping equipment or any vacuum driven setup other than gravity. The permeation water fluxes were approximately 25 and 17 $\frac{L}{{m}^{2}.h}$ at the end of 1 h through the CA membranes without and with PCL NPs, respectively, and reduced to 15 and 11 $\frac{L}{{m}^{2}.h}$, respectively, at the end of 8 h of operation (Fig. 9a). These results were in agreement with previously reported values^91–93^. Although the incorporation of PCL NPs appeared to create larger in size pores in the membrane structure (Figs 9a and 9b) that could be favorable for water to flow through, it also increased the membrane thickness and hence the overall mass transfer resistance to water flow quite significantly, which may explain the resultant lower permeation fluxes. In addition, the presence of NPs occupying the pore space could also have a similar effect by resulting in significantly narrowed passageways for water flow. When LPS was mixed with water, the water fluxes were observed to be lowered as well (Fig. 9b). Specifically, the LPS-containing water fluxes at the end of 1 h and 8 h were reduced to ~5.4 and ~2.5 $\frac{L}{{m}^{2}.h}$ using the original CA membrane, and ~4.2 and ~2.2 $\frac{L}{{m}^{2}.h}$ using the CA membrane embedded with PCL NPs. There could be a number of factors contributing to this phenomenon, which were considered not within the scope of this work but worthy of future studies. For example, the binding of LPS, being large elongated molecules, to the surfaces of the pores and PCL NPs could significantly reduce the pore sizes for water flow. The addition of LPS also changed the mass density of the solution which would certainly affect the gravity-driven flow through the membrane. These factors can be pursued in the future in order to obtain a deeper understanding and enable further optimization of the membrane pore structure for achieving even greater processability of the LPS-containing solutions.

### CA membranes without and with PCL NPs for removing LPS from water

**Figure 10:**
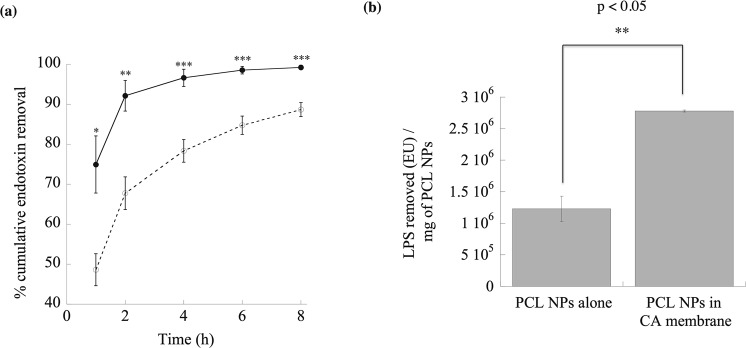
The LPS removal efficiency of PCL NP embedded filters. (**a**) Efficacy tests of CA membrane (open circles; ○) and CA membrane with PCL NPs (filled, solid circles; •) for the removal of LPS from the water. ${C}_{0}=270\frac{\mu g}{ml}\,LPS$ and PCL dose $\approx 1670\,\,\mu g/c{m}^{2}$of membrane. *, ** and *** indicate p values of 0.03, 0.01 and less than 0.005 respectively, demonstrating statistically significant differences between PCL NPs in CA membrane and CA membrane. (**b**) Bar plot of LPS removed (EU)/mg of PCL NPs in powder form and also in CA membrane. The extent of error bar for PCL NPs in CA membrane is small due to the fact that the percentage LPS removal reached ~100%. The difference between PCL NPs in powder and in the membrane is statistically significant (p < 0.05).

To confirm the adsorption capability of PCL NPs in a membrane form for potential application in larger scale operations, the LPS removal efficiencies by the CA membranes with or without PCL NPs were measured and compared. As can be seen in Fig. 10a, the incorporation of PCL NPs in membrane significantly boosted the LPS removal efficiency from ~48% to ~75% at the end of 1 h, and from 88% to near completion at the end of 8 h. The specific endotoxin units (EU) removed were further calculated and compared in Fig. 10b and Table S9, which clearly demonstrated the superior performance of PCL NPs in the membrane as compared to its pristine powder form. The removal efficiency per unit area was $\sim 4.3\times {10}^{4}$ EU/cm^2^ (~$2.8\times {10}^{6}$ EU/mg of PCL NPs), which was 2-fold (p < 0.005) higher than that of NPs alone (Table S9). These results indicate a promising avenue for removing LPS without the requirement of any pumping devices or external power sources through the utilization of PCL NPs both in powder and membrane forms.

## Product Comparison

**Figure 11:**
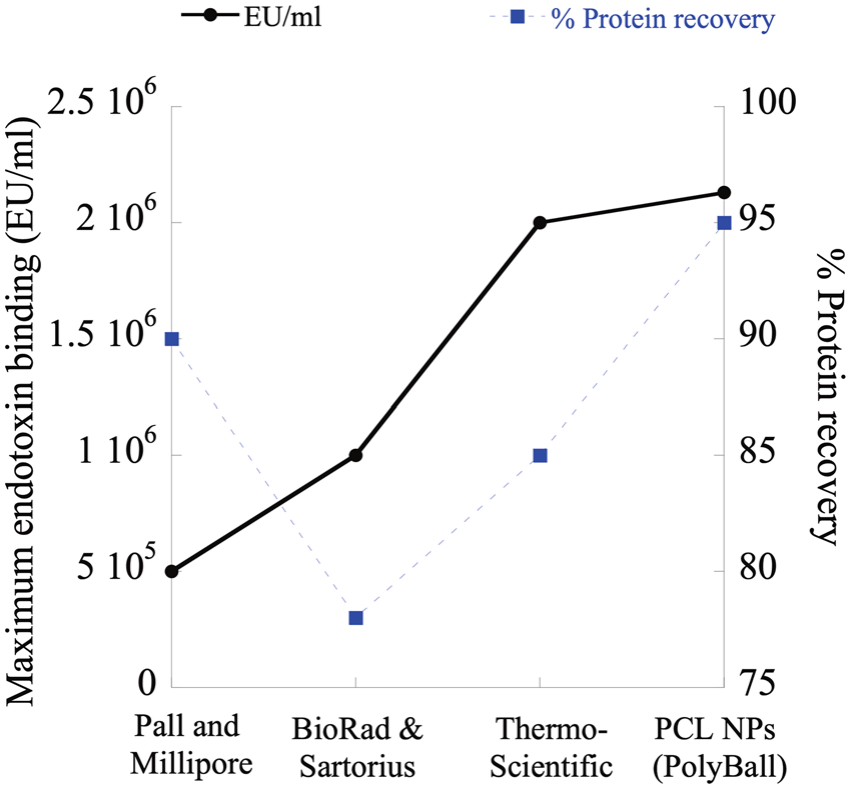
Product comparison. PCL NPs show higher LPS binding capacity as well as higher protein recovery than five commercially available endotoxin removal products.

PCL NPs and PCL NP retaining membranes were compared against five commercially available endotoxin removal products (Fig. 11 and Table 1) following the manufacturers’ instructions. A neutral pH 7.4 PBS solution containing ~2.8 ×10^6^ EU/ml of endotoxin was loaded in the presence of each product to determine the LPS clearance and protein recovery. PCL NPs and membranes showed 1.25 to 30-fold higher efficiency than other commercially available products.

**Table 1:** Comparison of PCL NPs and the NP containing membrane versus four commercially available endotoxin removal products.

Product Name	Maximum Endotoxin Binding capacity (EU/ml)	Cost ($)	Reusability
Pall Acrodisc Unit with Mustang E membrane	5.0 × 10^5^	$ 9.2 per 1 cm^2^ membrane area	Yes
Millipore charged Durapore cartridge membrane filters	>5.0 × 10^5^	$ 2.7 per 1 cm^2^ membrane area	Yes
BioRad Proteus Endotoxin Removal Kits (Membrane based)	5.0 × 10^5^ − 10^6^	$ 12.4 per 1 cm^2^ membrane area	Yes
Sartobind Q100 membrane adsorbers (Sartorius)	1.0 × 10^6^	NA	Yes
Thermo scientific Pierce High capacity endotoxin removal resins	2 × 10^6^	$ 20.2 per ml of resin slurry	Yes
PCL nanoparticles	1.45 × 10^6^	$2.4 per 1 g	Yes
PCL nanoparticles incorporated membrane	2.8 × 10^6^	$ 0.05 per 1 cm^2^ membrane area	Not tested yet

## Discussion

Relatively few polymers have been investigated for their potential to be synthesized into NP adsorbents for LPS removal. On a preparative scale, an important indicator of desirable properties from such NP adsorbents is the adsorption capacity per unit mass. In this work, the equilibrium adsorption capacity of PCL NPs in powder form and in the membrane was found to be more than $2.8\times {10}^{6}EU/mg$ of NPs as shown in Tables S2–S6 and Table S9. Previously, polymyxin B cross-linked cellulose porous microspheres of ~150 μm in diameter have been shown to have a maximum adsorption capacity of 3.6 × 10^6^ EU/mg^77^. These porous beads, despite offering a high internal surface area for LPS adsorption, also present hindered intraparticle mass transport within their porous structure so that their use in a membrane or in a chromatographic column requires a large pressure drop^94^. One way to circumvent this challenging issue of high pressure drop associated with high internal adsorption capacity is to use a nonporous solid adsorbent particle that has sufficient capacity on the exterior surface to achieve high adsorption efficiency at short residence time and under low pressure drop. As a type of such desirable adsorbent particles, PCL NPs of ~780 nm in diameter have a BET specific area of $\approx 6.5\,{m}^{2}/g$ that provides 82–98% LPS removal efficiency in water and PBS. These data are comparable to other previously reported processes^46,77,79^ and indicative of the potential of PCL NPs to fill the gap as a suitable adsorbent for LPS removal.

The extent of LPS removal was found in previous studies to depend on the characteristics of the buffer solution, including salt concentration and pH. Increasing the ionic strength was found to enhance the LPS adsorption on Q-sepharose gel column^67^. The LPS adsorption levels were 10^2^ and 10^3^ EU/ml in 10 and 50 mM PBS, respectively^67^. Similar high LPS binding properties were shown by hydroxyapatite, polystyrene, Dowex 1-X2, activated charcoal, phenyl- and octyl-sepharose in presence of a high concentration of ammonium sulfate salts^69^. Our PCL NPs were found to remove more than 10^6^ EU/ml using 150 mM PBS containing 137 mM NaCl (Figs 2–5), which represents an adsorption level almost 1000 fold higher than those of the previously published results. The effects of pH (protons) are also contingent on the electrostatic properties of the adsorbents^68,95^. In this work, the adsorption driving forces between the generally hydrophobic PCL NPs (adsorbent) and LPS (adsorbate) are dominated by the van der Waals interactions and hydrophobic binding, which are further enhanced by increasing pH that weakens the repulsion between the adsorbent and the adsorbate as both possess partially negatively charged moieties. The enhancement in LPS binding to hydrophobic PCL surface can be attributed to the weakening of the shielding effect common with water molecules which cannot wet the hydrophobic surface and instead form highly ordered shell-like structure or shield around the hydrophobic surface due to its inability to form hydrogen bonds in all directions, thus enhancing the interaction between two hydrophobic surfaces (LPS and PCL)^66–71^.

Combinedly, our results suggest that the highly effective LPS separation could be due to synergistic van der Waals and hydrophobic-hydrophobic interactions driving the selective LPS binding with the PCL NP surface. The hydrophobic interaction of LPS lipid tails with PCL NPs allows recruitment and assembly of LPS molecules on the NP surface. This process is synergized further due to the hydration of LPS polar head groups by the partially positively charged hydrogen ions of water. When LPS and PCL NPs are introduced to a protein solution, water molecules may rearrange by forming hydrogen bonds surrounding the LPS-PCL nanoparticle complex shell, thus effectively secluding the access of proteins to the particles. Because of this unstable nature of partial hydrogen ion plane surrounding the LPS-PCL NP complexes as well as individual observations, a wide variation in standard deviation was measured in water. In contrast, the presence of lyotropic salts like sodium chloride in PBS interacts strongly with these water molecules thus leaving less water available for the shielding effect to take place.

The effect of different buffers at variable pH’s and constant ionic strength was investigated (Fig. 6). Isoelectric point for LPS is at pH 2, hence LPS is negatively charged at pH > 2^80^. PCL NPs, on the other hand, has an isoelectric point at around pH 4^96^ and thus are positively charged at pH < 4 and negatively charged for pHs > 4. At pH 2.8 (acetic acid buffer), LPS would be negatively charged and PCL will have a positive charge, hence in addition to strong hydrophobic and van der Waals interaction, ionic interaction contributes towards LPS binding on PCL and thus a high LPS removal of ~90% was observed. The presence of acetate ion (CH_3_COO^−^) which is a lyotrope also helps in enhancing or promoting the hydrophobic interaction even further. As the buffer pH increases greater than 4, both PCL NPs and LPS exhibit negative charges due to their carbonyl and phosphate groups respectively. Based on these results, it can be concluded that in case of phosphate buffer (pH 5.8–8) the repulsion between LPS and PCL NPs dominates the hydrophobic and van der Waals interactions and therefore results in reasonably low LPS removal efficiency varying between 30–75%. For sodium bicarbonate buffer (pH 9.6), there was a sharp rise in LPS removal efficiency up to ~90%, indicating that the hydrophobic and van der Waals interaction dominates the repulsion action between PCL and LPS molecules at high pH. One major advantage of the biocompatible PCL particles is that they can be reused for LPS binding quite effectively without a major loss in binding efficiency (Fig. 7).

The LPS removal efficiency is further increased when PCL NPs were incorporated into a CA membrane, resulting in an adsorptive membrane that delivers a productivity flowrate of up to 25 $\frac{L}{{m}^{2}.h}$ (Fig. 9a,b)^97^. The porous CA membrane structure (Fig. 8a) has a small thickness (Table S6) and a favorable pore size distribution to not require high pressure drops for water flow across the membrane. Further insight in this respect can be obtained from an analogy using the Hagen-Poiseuille equation,

7 $${\rm{\Delta }}P=\frac{\mu Lq}{2\pi {a}^{2}}$$

where the pressure difference ($${\rm{\Delta }}P$$) can be related to $$\mu =viscoty\,of\,water=8.9\times {10}^{-4}Pa\cdot s$$, $$L={\rm{membrane}}$$$${\rm{thickness}}=130\times {10}^{-6}m$$, $$q=volumetric\,\,flow\,rate=25\frac{L}{{m}^{2}.h}=6.9\times {10}^{-6}m/s\,$$ and $$\,a=pore\,diameter=$$$$0.17\,\times {10}^{-6}m$$. The resultant $${\rm{\Delta }}P$$ is equivalent to a low value of 63 Pa, which confirms the unnecessity of any pumping device for the solution to pass through the membrane to allow the adsorption removal of LPS to take place on the inside by the PCL NPs.

It is worth mentioning here that one direction for future study is to optimize the membrane pore structure to achieve higher productivity flowrates without sacrificing the loading and adsorption capability of PCL NPs. Some possibilities^98^ in this regard could result from using more branched cellulose polymers, additives or cross-linkers, and templated casting surface. In addition, a very preliminary cost analysis was performed (Table 1) to get an idea of the costs associated with manufacturing the PCL NP embedded CA membrane. The result was acceptably less than a dollar per cm^2^. However, more extensive and rigorous analysis is needed when an actual process is being designed or in operation, which needs to take into account labor, utilities, storage, and other process variables including potentially pumping devices.

## Conclusion

In this study, we report first the synthesis of polymeric PCL NPs by employing a solvent evaporation method and then the performances of PCL NPs for the adsorption and removal of LPS. It was found that PCL NPs in powder form removed around 88% of LPS from the water sample. The presence of salts *via* the addition of PBS increased the LPS removal efficiency further up to 100% by PCL NPs, while maintaining 100% protein recovery from solutions. This high removal efficiency of LPS from water and PBS attributed to strong hydrophobic and van der Waals interaction. Buffers of variable pH play a very important role in determining the LPS binding on PCL. Acidic (pH 2.8) and alkaline (pH 9.6) buffers give ~90% LPS removal whereas intermediate pHs from 5.8 to 8 give reasonably lower % LPS removal between 30–75%. The adsorption efficiency reached almost 100% when PCL NPs were incorporated into the CA membrane where the water flow through the porous structure was directly by gravity without the requirement of any pumping devices. The biocompatible PCL NPs can be reused by desorbing majority of adsorbed LPS using 0.2 N NaOH solution. A preliminary cost analysis showed that the manufacturing cost of the PCL NP embedded CA membrane is quite affordable. These findings coupled with PCL NP’s known biodegradability support the potential of hybrid NP-membrane system to be used in large-scale operations that remove LPS efficiently and reduce the downstream process costs in biotechnological industries.

## Supplementary information

Supplementary Information
