# Supplementary material for: PolyBall: A new adsorbent for the efficient removal of endotoxin from biopharmaceuticals
Source: Sci Rep. 2019 Jun 20;9:8867. doi: 10.1038/s41598-019-45402-w (PMC6586805; doi:10.1038/s41598-019-45402-w)
Supplement: Supplementary file 1 — Supplementary Information [file 41598_2019_45402_MOESM1_ESM.pdf]

Supplementary Information

PolyBall: A new adsorbent for the efficient removal of endotoxin from biopharmaceuticals

Sidharth Razdan, Jee-Ching Wang, and Sutapa Barua[1]

Department of Chemical and Biochemical Engineering

Missouri University of Science and Technology, Rolla, MO 65409

[1] To whom correspondence should be addressed. Email: baruas@mst.edu. Department of Chemical & Biochemical Engineering, 110 Bertelsmeyer Hall, 1101 N. State Street, Rolla, MO 65409-1230

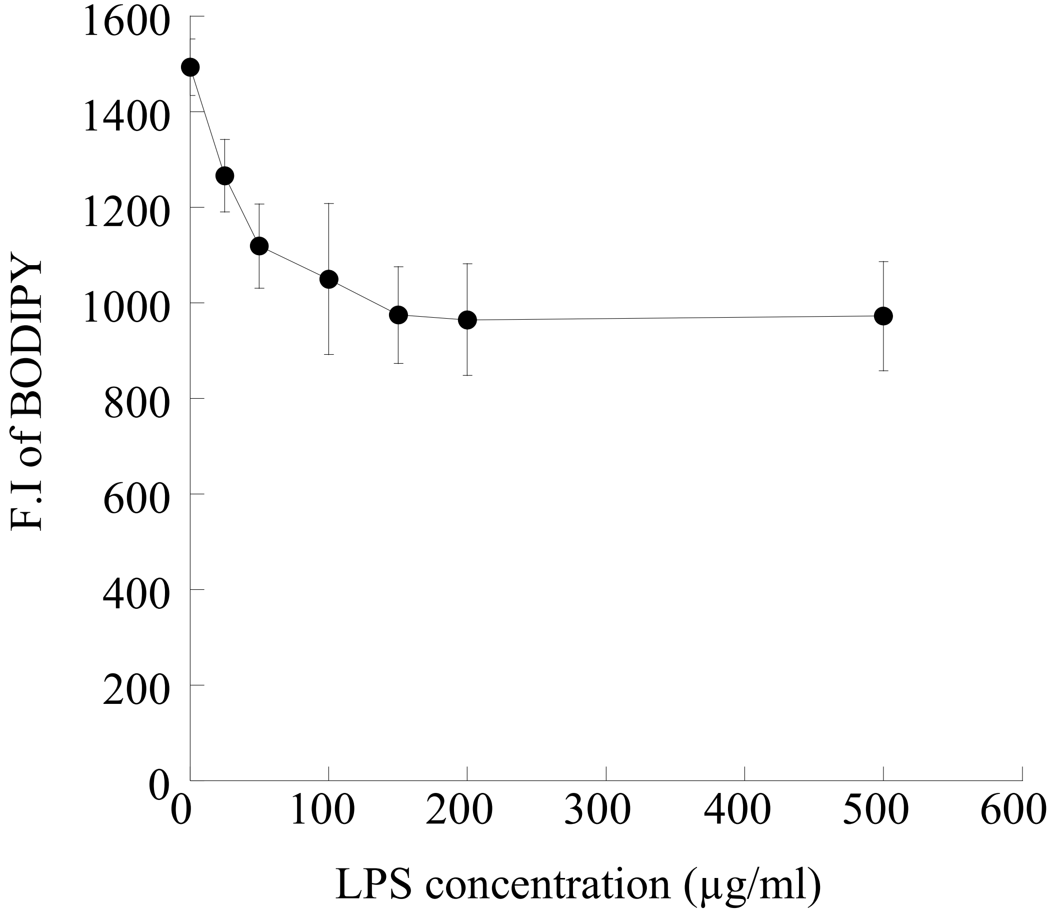

Figure S1: A standard curve to determine the optimum mass ratio of BODIPY : LPS interaction at different concentrations of LPS. BODIPY concentration was 262.11μg/ml. Addition of LPS to BODIPY results in quenching due to binding of LPS to BODIPY sites. The binding saturates at 150 μg/ml endotoxin concentration. The optimum ratio of BOD: LPS was found to be 1.74.

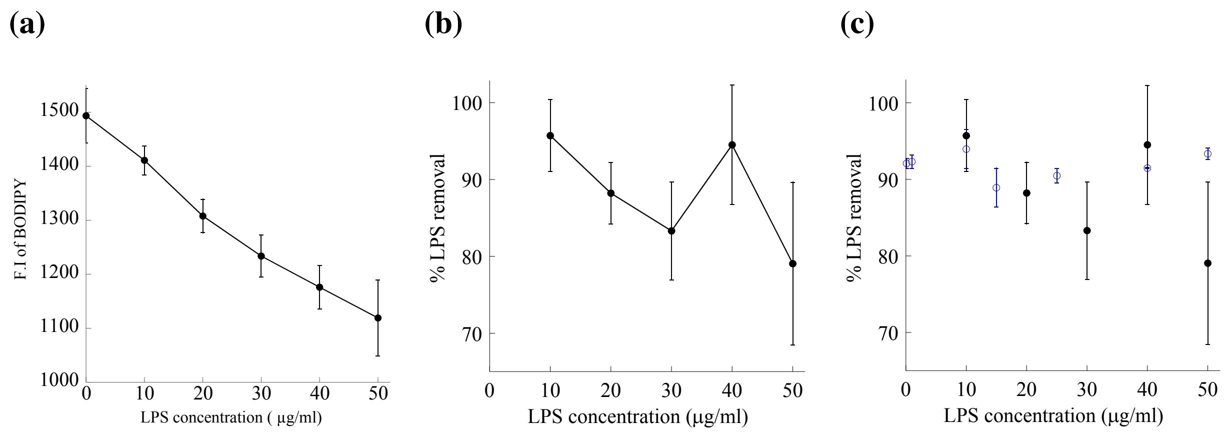

Figure S2: To confirm the linear correlation of the BODIPY assay standard curve for LPS detection (Figure S1) within the range of 0-50 μg/ml, the calibration curve was regenerated independently over the range of interest. **(a)** The BODIPY assay standard curve indeed shows a linear relationship at this low LPS concentration range. We further tested this new standard curve to calculate % LPS removal using LPS feed concentrations between 0-50 μg/ml. **(b)** The % LPS removal calculated using the correlation shown in **(a)**. **(c)** The % LPS removal (solid circles) was compared with the values generated using Figure S1(open circles) as shown in Figure 2 (b) in the main texts. The values from two independently generated standard curves were found to be evenly distributed and fit within the error range which ensured the preparation of the calibration standards.

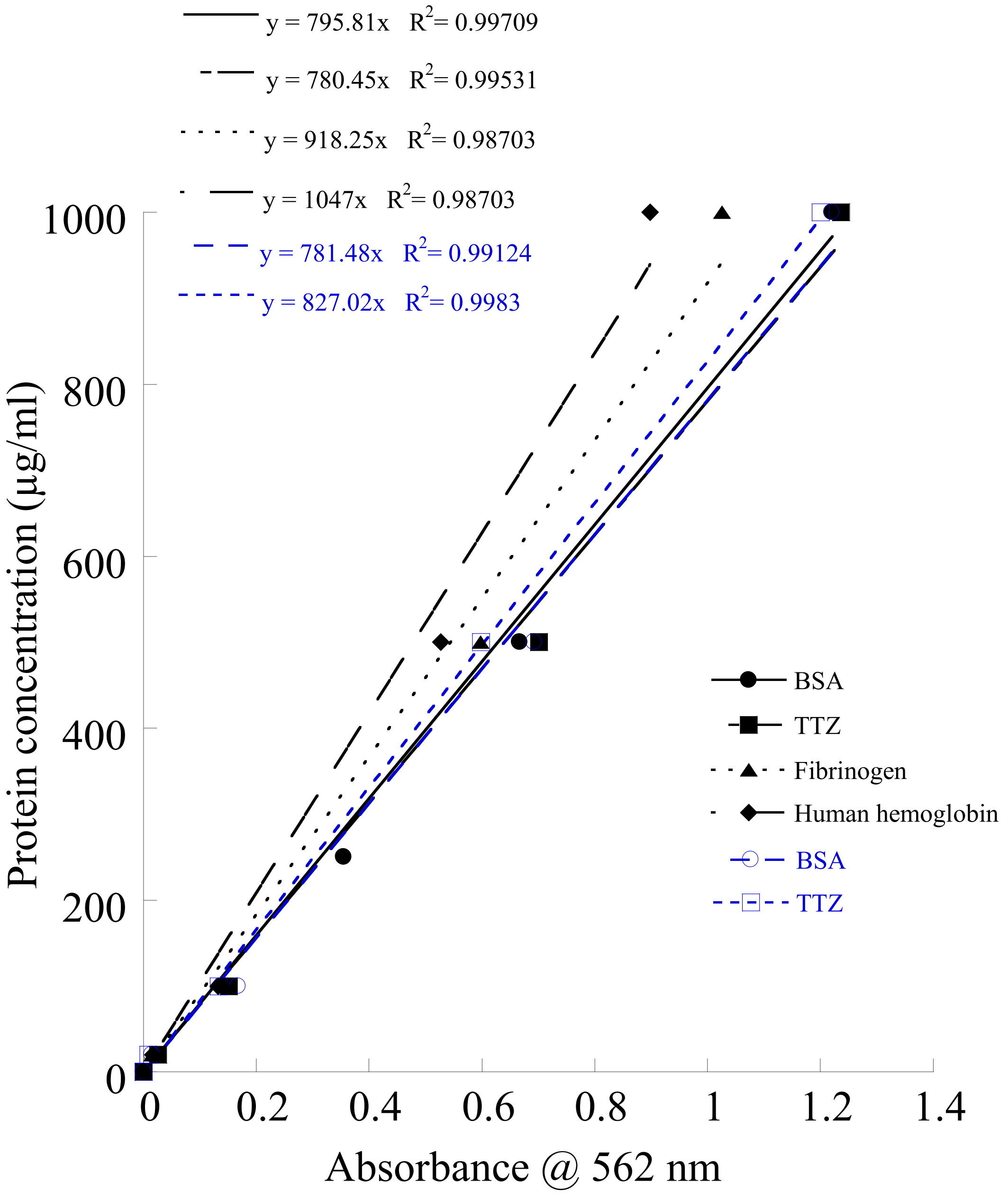

Figure S3: Protein standard curves of BSA and Trastuzumab (TTZ; Genentech) in water and PBS (pH 7.4) and Fibrinogen and Human hemoglobin in PBS (pH 7.4) were used to measure protein concentrations. R^2 represents the regression value

Table S1: Buffer recipes of different pHs to study the effects of pH on LPS removal

**Buffer type (pH); ionic strength**	**Recipe**
Glacial acetic acid buffer (2.8) ; 0.1M	Mix 5.72 ml of glacial acetic acid, 994.28 ml of DI water and 300 μl of concentrated hydrochloric acid
Phosphate buffer (5.8) ; 0.1M	Mix 4 ml of 0.2 M sodium phosphate dibasic dihydrate ($Na_2HPO_4 \cdot 2H_2O$, FW=178.05) in water with 46 ml of 0.2 M sodium phosphate monobasic monohydrate ($NaH_2PO_4 \cdot H_2O$, FW=138.01) in water
Phosphate buffer (6.8) ; 0.1M	Mix 24.5 ml of 0.2 M sodium phosphate dibasic dihydrate ($Na_2HPO_4 \cdot 2H_2O$, FW=178.05) in water with 25.5 ml of 0.2 M sodium phosphate monobasic monohydrate ($NaH_2PO_4 \cdot H_2O$, FW=138.01) in water
Phosphate-buffered saline PBS (7.4) ; 0.1M	Mix 20 ml of 0.150 M PBS solution (137 mM NaCl, 10 mM phosphate, and 2.7 mM KCl) with 10 ml of DI water to obtain 30 ml 0.1 M PBS (91.4 mM NaCl, 6.7 mM phosphate, and 1.9 mM KCl)
Phosphate buffer (8.0) ; 0.1M	Mix 47.35 ml of 0.2 M sodium phosphate dibasic dihydrate ($Na_2HPO_4 \cdot 2H_2O$, FW=178.05) in water and 2.65 ml of 0.2 M sodium phosphate monobasic monohydrate ($NaH_2PO_4 \cdot H_2O$, FW=138.01) in water
Sodium bicarbonate buffer (9.6) ; 0.1M	Mix 8.4 g of sodium bicarbonate ($NaHCO_3$) with 991.6 ml of DI water

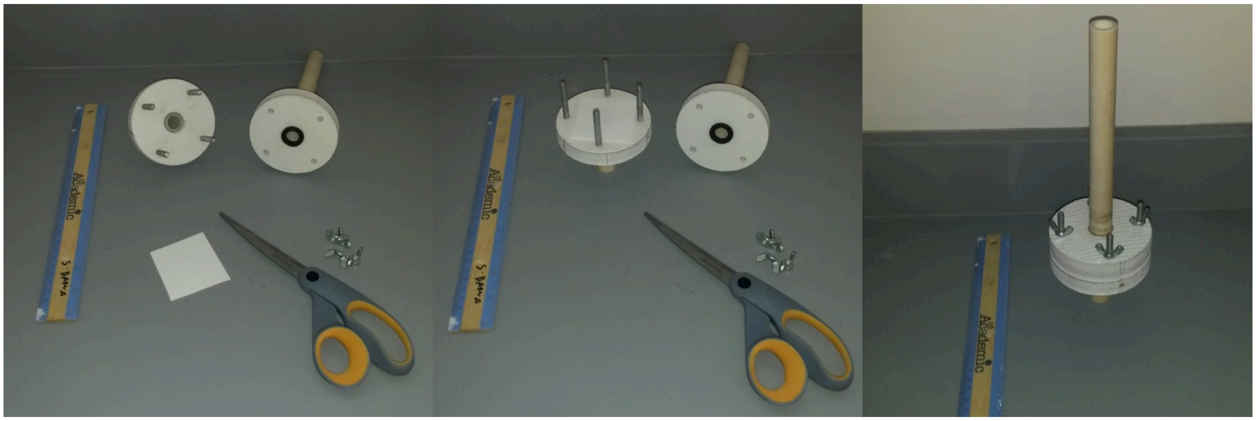

Figure S4: Experimental set up of a custom-made PVC column to sandwich CA membrane without and with PCL NPs in between two flow pipes. A piece of membrane was screw tightened between two flow pipes (top and bottom). Water and LPS containing water was fed on the top PVC pipe and let it flow by gravity. The permeability of membrane and % LPS removal were assessed using this set-up. A video on how samples were collected is shown in SI movie 1.

Table S2: The LPS binding capacity, endotoxin unit (EU) removed by per surface area and per milligram of PCL NPs in water. 1 EU$\approx 0.1 - 0.2\ ng$ of LPS

LPS fed (endotoxin unit; EU)	PCL NP concentration (mg/ml)	PCL density (g/ml)	PCL NP diameter (μm)	# of PCL NPs/ml	PCL NPs' surface area/ml (cm^2/ml)	LPS removed (EU) /cm^2 NPs	LPS removed (EU) /mg of PCL NPs
1.5×10^6	1	1.1	0.7	3.3×10^9	65	20.4×10^3	1.3×10^6
1.25×10^6						16.2×10^3	1.0×10^6
1.0×10^6						12.4×10^3	0.8×10^6
0.75×10^6						9.8×10^3	0.6×10^6
0.5×10^6						7.1×10^3	0.5×10^6
4.0×10^5						5.6×10^3	3.7×10^5
2.5×10^5						3.0×10^3	2.3×10^5
1.5×10^4						2.0×10^3	1.3×10^5
1.0×10^5						1.45×10^3	9.4×10^4
1.0×10^4						1.4×10^2	9.2×10^3
1.0×10^3						14.0	9.2×10^2

Table S3: The LPS binding capacity (EU/cm² and EU/mg) by PCL NPs from varying BSA concentrations in PBS (pH 7.4) when treated with a fixed concentration of LPS (150 μg/ml ≈ 1.5×10^6 EU/ml). 1 EU≈ $0.1 - 0.2\ ng$ of LPS

BSA concentration (μg/ml)	PCL NP concentration (mg/ml)	PCL density (g/ml)	PCL NP diameter (μm)	# of PCL NPs/ml	PCL NPs' surface area/ml (cm²/ml)	LPS removed (EU) /cm² NPs	LPS removed (EU) /mg of PCL NPs
250	1	1.1	0.7	3.2×10^9	65	21.0×10^3	1.4×10^6
500						21.5×10^3	1.4×10^6
1000						22.0×10^3	1.4×10^6

Table S4: LPS binding capacity (EU/cm² and EU/mg) by PCL NPs from varying Trastuzumab concentrations in PBS (pH 7.4) when treated with a fixed concentration of LPS (150 μg/ml ≈ 1.5×10^6 EU/ml)

TTZ concentration (μg/ml)	PCL NP concentration (mg/ml)	PCL density (g/ml)	PCL NP diameter (μm)	# of PCL NPs/ml	PCL NPs' surface area/ml (cm²/ml)	LPS removed (EU) /cm² NPs	LPS removed (EU) /mg of PCL NPs
250	1	1.1	0.7	3.2×10^9	65	22×10^3	1.4×10^6
500						22×10^3	1.4×10^6
1000						22×10^3	1.4×10^6

Table S5: LPS binding capacity (EU/cm² and EU/mg) by PCL NPs from varying Fibrinogen concentrations in PBS (pH 7.4) when treated with a fixed concentration of LPS (150 μg/ml ≈ 1.5×10^6 EU/ml)

**Fibrinogen concentration (μg/ml)**	**PCL NP concentration (mg/ml)**	**PCL density (g/ml)**	**PCL NP diameter (μm)**	**# of PCL NPs/ml**	**PCL NPs' surface area/ml (cm²/ml)**	**LPS removed (EU) /cm² NPs**	**LPS removed (EU) /mg of PCL NPs**
250	1	1.1	0.7	3.2×10^9	65	21×10^3	1.4×10^6
500						20×10^3	1.3×10^6
1000						21×10^3	1.4×10^6

Table S6: LPS binding capacity (EU/cm² and EU/mg) by PCL NPs from varying Human hemoglobin concentrations in PBS (pH 7.4) when treated with a fixed concentration of LPS (150 μg/ml ≈ 1.5×10^6 EU/ml)

**Human Hemoglobin concentration (μg/ml)**	**PCL NP concentration (mg/ml)**	**PCL density (g/ml)**	**PCL NP diameter (μm)**	**# of PCL NPs/ml**	**PCL NPs' surface area/ml (cm²/ml)**	**LPS removed (EU) /cm² NPs**	**LPS removed (EU) /mg of PCL NPs**
250	1	1.1	0.7	3.2×10^9	65	21×10^3	1.3×10^6
500						19×10^3	1.2×10^6
1000						19×10^3	1.2×10^6

Table S7: Comparison of adsorption isotherms of LPS removal with literature values

LPS binding material	LPS Feed range (EU/ml)	Langmuir isotherm parameters			Freundlich isotherm parameters		
		q_m (μg LPS/mg)	K (ml solution/ mg)	R^2	K (μg LPS/mg PCL NPs)	n	R^2
PCL NPs	$10^3 - 5 \times 10^6$ (in water)	-	-	-	9.5	1.1	0.95
PCL NPs	$10^4 - 1.5 \times 10^6$ (BSA in water)	-	-	-	7.6	1.3	0.99
PCL NPs	$10^4 - 1.5 \times 10^6$ (IgG in water)	-	-	-	8.7	1.4	0.93
PCL NPs	$10^4 - 1.5 \times 10^6$ (BSA in PBS)	-	-	-	13	1.2	0.99
PCL NPs	$10^4 - 1.5 \times 10^6$ (IgG in PBS)	-	-	-	11.7	1.3	0.98
PMB-CL-CMs[1]	1–100	3.6×10^{-4}	6.5×10^6	0.99	-	-	-
Si gel-His[2]	$10^3 - 6 \times 10^4$	1.2	740	0.90	-	-	-
Agarose[3]	$10 - 1.8 \times 10^7$	3300	56	0.99	-	-	-
Bone Char[4]	1–100	2.9×10^{-6}	9.0×10^{-8}	0.99	-	-	-

PMB-CL-CMs= Polymyxin B immobilized cross-linked cellulose microspheres

Si gel-His= Histidine immobilized silica gel

(a) The surface charge of poly-l-lysine (PLL) coated PCL NPs

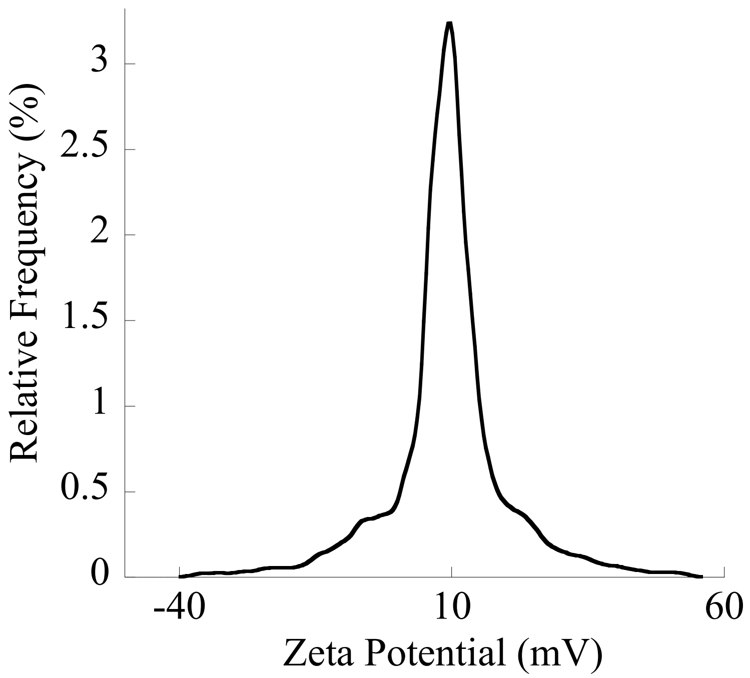

(b) Comparison of % LPS removal without and with PLL coated PCL NPs

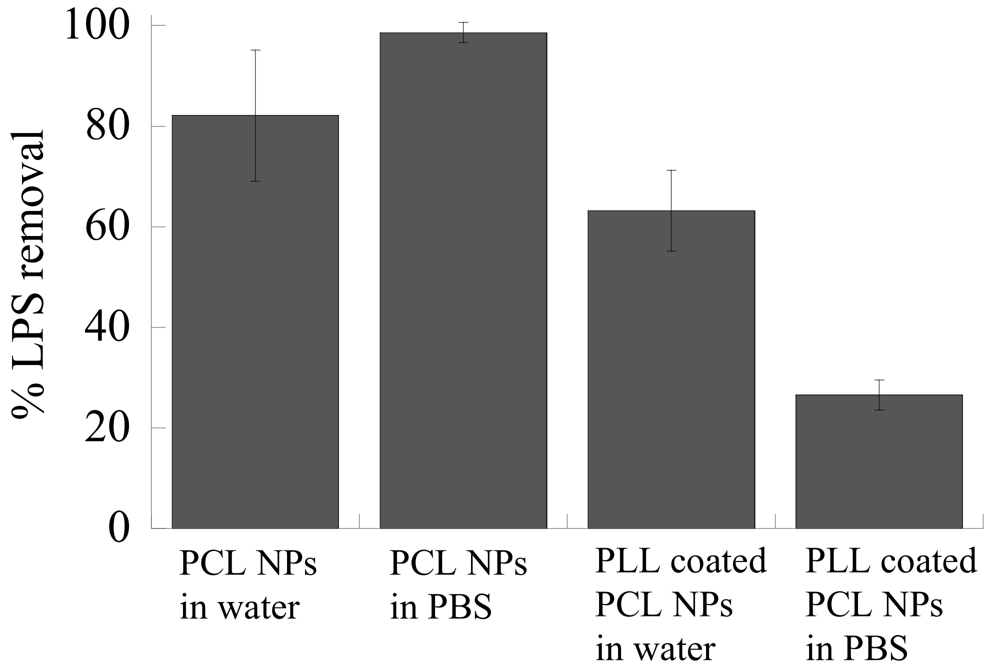

Figure S5: **(a)** The surface zeta potential of positively charged poly-l-lysine (PLL) coated PCL NPs which confirmed the clear shift in surface charge towards (10 ± 0.3) mV from (-20 ± 5) mV

of bare PCL NPs (Figure 1(c)). **(b)** The PLL coated PCL NPs showed ~20 and 80% reduction in % LPS removal in water and PBS, respectively compared to bare PCL NPs indicating the cationic charges on the surface of PCL NPs decreased the interaction of LPS with the NP surface.

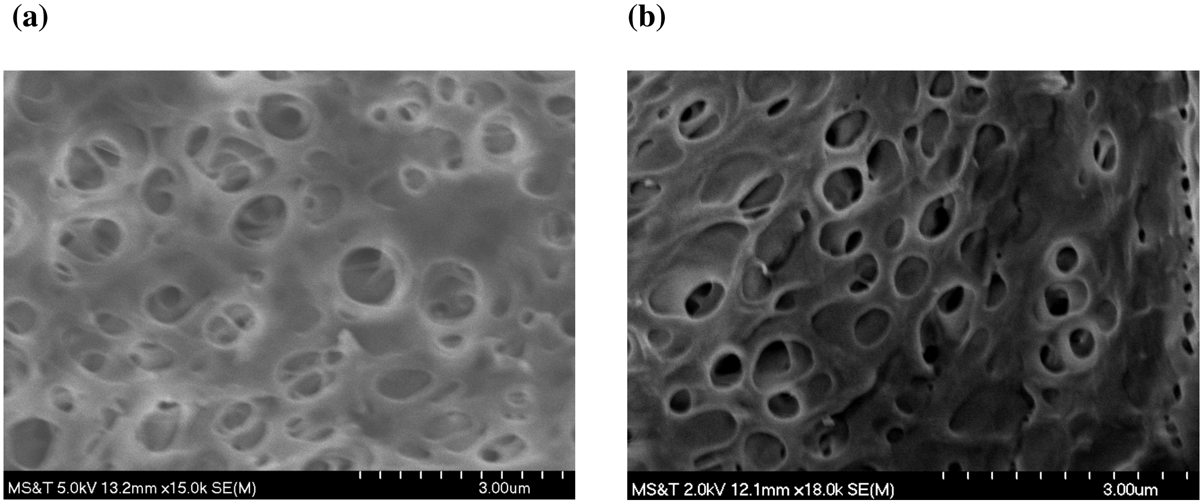

Figure S6: Surface SEM morphology of CA membrane **(a)** without and **(b)** with PCL NPs

Table S8: Calculations of CA membrane thickness and pore size with and without PCL NPs

**Membrane type**	**Membrane thickness (μm ± S.D.)**	**Surface pore diameter (μm ± S.D.)**
CA Membrane	116 ± 2	0.16 ± 0.05
CA Membrane with PCL NPs	132 ± 12	0.17 ± 0.05

Table S9: Detailed calculations of EU removed by PCL NPs impregnated in CA membrane

PCL NPs present in the membrane (mg)/membrane	Endotoxins fed (endotoxin unit; EU)	LPS removed (EU)/cm^2	LPS removed (EU)/mg of PCL NPs
3.0 ± 1.2	8.5×10^6	42.6×10^3	2.8×10^6

References

1. Cao, X., Zhu, B., Zhang, X. & Dong, H. Polymyxin B immobilized on cross-linked cellulose microspheres for endotoxin adsorption. *Carbohydrate Polymers* **136**, 12-18 (2016).
2. Zhang, Y., Yang, H., Zhou, K. & Ping, Z. Synthesis of an affinity adsorbent based on silica gel and its application in endotoxin removal. *Reactive and Functional Polymers* **67**, 728-736 (2007).
3. Wei, Z., Huang, W., Hou, G., Yuan, Z. & Fang, J. Studies on adsorption isotherms of endotoxin and BSA using an affinity column. *Process Biochemistry* **42**, 285-288 (2007).
4. Rezaee, A., Ghanizadeh, G., Behzadiyannejad, G., Yazdanbakhsh, A. & Siyadat, S.D. Adsorption of Endotoxin from Aqueous Solution Using Bone Char. *Bulletin of Environmental Contamination and Toxicology* **82**, 732-737 (2009).
